# Flexible Polyurethane Foams Reinforced by Functionalized Polyhedral Oligomeric Silsesquioxanes: Structural Characteristics and Evaluation of Thermal/Flammability Properties

**DOI:** 10.3390/polym14214743

**Published:** 2022-11-05

**Authors:** Edyta Hebda, Artur Bukowczan, Sławomir Michałowski, Krzysztof Pielichowski

**Affiliations:** Department of Chemistry and Technology of Polymers, Cracow University of Technology, Warszawska 24, 31-155 Krakow, Poland

**Keywords:** flexible polyurethane foam, oligosilsesquioxanes, hybrid materials, POSS, flammability

## Abstract

In this work, we report on flexible toluene diisocyanate (TDI)-based polyurethane foams (FPUFs) chemically modified by POSS moieties, i.e., octa (3-hydroxy-3-methylbutyldimethylsiloxy) POSS (OCTA-POSS) and 1,2-propanediolizo-butyl POSS (PHI-POSS). The influence of silsesquioxane on the PU foaming process, structure, morphology, physicochemical, and mechanical properties, as well as flammability, was examined. FT-IR analysis provided evidence for the chemical incorporation of the nanofiller into the foam structure. It was found that the addition of POSS increases the apparent density of the foam and its compressive strength. The XRD and SEM-EDS techniques showed the uniform distribution of POSS in the FPUF with agglomeration depending on the kind and content of the introduced POSS moieties. The analysis of the thermogravimetric and microcalorimetry data revealed an improved resistance to the burning of FPUFs containing reactive POSS, as evidenced by the reduced rate of heat release (HRR). Importantly, the mechanical properties tests showed that the incorporation of silsesquioxane nanoparticles into the polyurethane structure via covalent bonds strengthens the foam integrity.

## 1. Introduction

Although the development of polyurethanes (PUR) began more than 75 years ago, intensive works are still ongoing to improve the physicochemical parameters of this important class of polymeric materials. The undoubted advantage of polyurethanes is the possibility of producing them by using various methods and components, meaning that it is possible to design materials with the desired characteristics. The scope of PUR application is very wide, as by changing the raw materials, their mutual volume ratio, and selecting the processing conditions, it is possible to obtain both solid and porous materials [1]. Porous materials may present appropriate properties in a number of engineering applications [2]. Flexible polyurethane foams (FPUFs), one of the PUF materials with an open-cell structure, show much greater possibilities for modifying the physical and mechanical properties of the final products compared to solid materials, e.g., the apparent density can be changed in the range from 10 to 120 kg/m^3^, using the same production equipment [3]. Thus, FPUF has a wide range of industrial applications, including furniture, bedding, carpet underlay, automotive interiors, and packaging [4].

FPUF is usually obtained using a one-step method, in which, after intensive mixing and pouring all the raw materials into the mold, the foaming process occurs. Water plays an extremely important role in the production of FPUF since it allows the release of carbon dioxide, which is the main foaming agent [5,6]. The cell structure and the microphase behavior determine the properties of FPUF. The reaction of isocyanate with polyol leads to the formation of the PUR chain and network, and as a result of phase separation, rigid urethane segments and flexible polyol-derived segments are formed. The FPUF morphology at different magnification scales has an open-cell structure with cell sizes ranging from 0.1 to 1.0 mm. The value of the Young’s modulus of the foam is inversely proportional to the fourth power of the cell size and the square of the thickness of the struts, assuming a constant apparent density [7]. In fact, foam materials are closely relative to their structural parameters, especially porosity, in their properties [8]. The segmental FPUF microstructure, compared to PUR elastomers, is enriched with a urea segment, forming spherical aggregates with a size of approx. 0.3 μm. Polyurea aggregates are formed as a result of the hydrogen bonds occurring between the C=O and N-H groups. These aggregates support cell opening after FPUF growth [9,10,11]. Urethane and rigid urea segments form rigid domains when using glass transition temperatures (T_g_) above 200 °C, providing FPUF with strength and thermal stability. Flexible PUF segments form a spatial PUR network with T_g_ ranging from −50 to −70 °C, giving the foams softness and elasticity [12].

The constant desire to improve the properties of the obtained materials has led to the introduction of various types of additives into polymeric materials. One of the developed ideas to improve polymer properties is to chemically build nanoparticles into the polymer structure, thus obtaining an organic–inorganic hybrid material. Among various nanomaterials, polyhedral oligosilsesquioxanes (POSS) are unique nanoadditives, which, thanks to their structure, combine the benefits of inorganic and organic compounds. These nanoparticles, depending on their structure and the reactivity of the vertex groups, can be introduced into the main chain, form side chains, or constitute a node of the polymer network. The introduction of POSS has a considerable impact on the dynamics of the macrochain, which leads to changes in the glass transition temperature [13,14,15,16,17,18]. Hybrid polymeric materials, thanks to their unique properties, have been considered for industrial and technological applications, including microelectronics, optoelectronics, nanocomposites, and biocompatible materials sectors [19,20,21]. Polyurethane/POSS composites were found to show reduced flammability, lower thermal conductivity, and a higher temperature of thermal decomposition compared to pristine polymer [17,22,23,24].

In our previous study [25], we investigated the influence of POSS nanoparticles on macromolecular architecture and bioactivity of flexible polyurethane foams based on aliphatic hexamethylene diisocyanate (HDI). In this work, we focused on studying the effect of the addition of POSS particles on the structure and properties of novel flexible polyurethane foams synthesized with aromatic toluene 2,4-diisocyanate (TDI). The influence of the applied modifiers on the foaming process was determined, along with the selected physical, mechanical, and thermal properties. Because the chemically built-in POSS particles are known as flame retardant agents, [26,27,28] we have also investigated the flammability of FPUF/POSS materials.

## 2. Materials and Methods

### 2.1. Materials

Flexible polyurethane foams were synthesized using Rokopol F3600 polyether polyol, a glycerine-based block-statistic copolymer of ethylene oxide and propylene oxide acquired from PCC Rokita S.A., and toluene diisocyanate (TDI) (Sigma Aldrich). As auxiliary agents, silicone L-627 from Momentive Performance Materials Inc., Dabco^®^ T-9, BL-11 catalyst from Air Products, and water as a chemical blowing agent, were applied. As the polyurethane foam modifiers, 1,2-propanediolisobutyl POSS (PHIPOSS) and octa (3-hydroxy-3-methylbutyldimethylsiloxy) POSS (OCTAPOSS) were used, which were provided by Hybrid Plastics (Hattiesburg, MS, USA).

### 2.2. Preparation of Polyurethane Foams

FPUF were produced on a laboratory scale using a two-component system in a one-stage method, with the NCO to OH group equivalent ratio equal to 1. Component A was obtained by thoroughly and vigorously mixing Rokopol F3600, surfactant, catalyst, blowing agent, and—in the case of modified foams—the appropriate amount of POSS, and component B was TDI. Table 1 shows the formulation of the obtained FPUF/POSS foams. The detailed procedure was described in Ref. [28]. Components A and B were mixed and poured into an open mold before the growth process (about 1 h), then conditioned at room temperature for at least 24 h.

### 2.3. Methods

The foaming process was analyzed using the FOAMAT^®^ equipment. The analysis was based on the measurement of the changes in the dielectric polarization of the foamed mixture, as well as the temperature during the foaming process, which allows the determination of the reactivity of the system.

The chemical structure of the obtained PUR foams was analyzed by infrared spectroscopy (FT-IR) using a Nicolet iS5 spectrometer equipped with a diamond crystal in an attenuated total reflectance unit manufactured by Thermo Electron Corporation. For each material, the measurement was performed five times, and the obtained results were averaged using the Omnic computer program.

The microstructure of the foams was characterized by scanning electron microscopy (SEM) using a JEOL InTouch Scope JSM-6010LV microscope with energy-dispersive X-ray analysis capabilities, operating at 10 kV accelerating voltage.

Wide-angle X-ray diffraction (WAXD) investigations were performed by applying a Bruker D-Phaser diffractometer in the reflection mode. A standard Cu-Kα anode with wavelength λ = 1.54184 Å was used.

The apparent density of the foams was determined in accordance with ISO 845 standard. The mass of the samples was determined by means of analytical balance with an accuracy of 0.1 mg, and the volume—as a result of dimensioning of rectangular samples using a caliper, with an accuracy of 0.1 mm.

The compressive strength was determined using a Zwick/Roell Z005 testing machine, in accordance with ISO 844 standard, with up to 40% deformation of the sample; the deformation speed was 10 mm/min.

Thermogravimetric analysis was performed using a Netzsch TG 209 F1 Libra thermal analyzer to determine the thermal stability of the obtained foams modified by POSS. The samples (sample mass ca. 5 mg) were heated in an open corundum pan from 30 up to 600 °C at a heating rate of 10 °C min^−1^ under an inert atmosphere.

The Pyrolysis Combustion Flow Calorimetry (PCFC) technique, developed by Fire Testing Technology Ltd. (East Grinstead, UK), uses traditional oxygen depletion calorimetry. The sample is first heated at a constant rate of temperature rise of 1 deg/s in a pyrolyzer. The thermal decomposition products are swept from the pyrolyzer by an inert gas. After pyrolysis, the gas stream is mixed with oxygen and enters a combustor at 900 °C, where the products after decomposition are completely oxidized. The oxygen concentrations and the flow rates of the combustion gases are used to determine the oxygen depletion involved in the combustion process and the heat release, as well as the heat release capacity.

Pyrolysis was performed using a vertical micro-furnace pyrolyzer (Frontier Lab., PY-2020iD) equipped with Shimadzu GCMS-QP2020 under a flow of a He inert gas. The flash pyrolysis temperature was set to 350 °C for 10 s. For the peak identification of the pyrolysis products, based on their retention times, the NIST 17 library was used. The resulting pyrolyzates (ca. 0.2 mg) were separated by a stainless-steel capillary column (Frontier Lab. Ultra ALLOY-5) coated with dimethylpolysiloxane liquid phase under the following program: 40 °C (2 min hold)/200 °C (1 min hold)/280 °C (4 min hold). The first step was carried out with a heating rate of 40 °C/min and the second at 30 °C/min. For mass spectroscopy detectors, the following parameters were applied: an ion source temperature of 200 °C, an interface temperature of 230 °C, and a detector voltage of 0.1 kV, with a range of *m/z* scan from 40 to 400.

## 3. Results

### 3.1. Foaming Process

**Figure 1:**
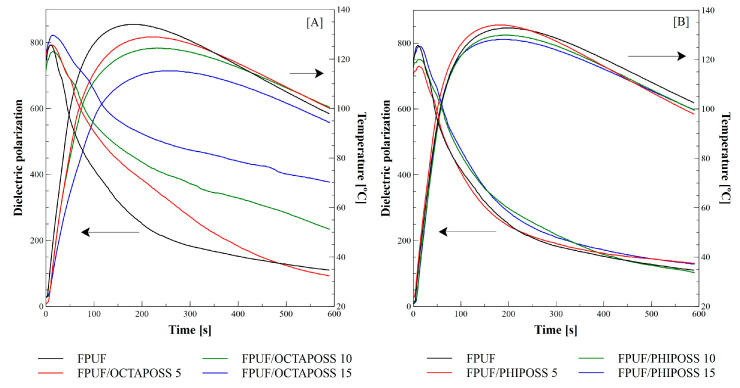
Influence of POSS addition to the reference system on dielectric polarization and temperature during the FPUR foaming process. (**A**) System FPUF/OCTAPOSS and (**B**) System FPUF/PHIPOSS.

Controlling the foaming process includes the control and measurement of different parameters, such as the temperature of the process and dielectric properties. It has been found that rapidly exothermic foaming reactions take place in the formulas synthesized with TDI isocyanate, as evidenced by the prompt increase in temperature up to its maximum value (Figure 1).

The highest temperature value was achieved for the FPUF reference material, in which the core temperature was 134 °C during the 210 s. It was observed that the type and amount of POSS additive introduced into the system reduced the maximum temperature during the foaming process compared to the reference foam. The addition of OCTAPOSS significantly reduces the core temperature, and in the case of its maximum content (15%wt.), the temperature drops to 115 °C in 253 s. This tendency is also observed for foams with the addition of PHIPOSS; however, the core temperature drop, in this case, is much smaller and amounts to 128 °C during 193 s. This difference results from the different structures of the introduced POSSs and their reactivity. The parameter presented in Figure 1 is the dielectric polarization related to the -OH and -NCO groups present in the mixture and their mobility. The cross-linking of the material leads to the limitation of the mobility of the functional groups; therefore, the dielectric polarization value decreases as the reaction progresses. From the value of dielectric polarization, one can conclude about the degree of conversion of the substrates. The intensive course of the foaming reaction in the FPUF/PHIPOSS system is confirmed by a sharp decrease in the dielectric polarizability value, as can be seen in Figure 1B. After 193 s (for FPUF/PHIPOSS), the dominant intensive reactions in the first stage of foaming are completed, as evidenced by the inflection point of the curve on the temperature diagram and a decrease in dielectric polarizability. When the temperature fluctuates at a relatively constant level, and the dielectric polarizability slightly decreases, which is illustrated by the flattening of the profiles, gelling processes take place. Gelation reactions take longer, as indicated by the long and delicate flattening of the curves. A higher value and a slight decrease in dielectric polarizability are observed for the FPUF/OCTAPOSS system—Figure 1A—which proves the higher electrical conductivity of the reaction mixture, i.e., a lower degree of conversion. This confirms the lower reactivity of the OCTAPOSS used. These materials achieve higher values and a more delicate decrease in dielectric polarizability.

### 3.2. Structure and Morphology

**Figure 2:**
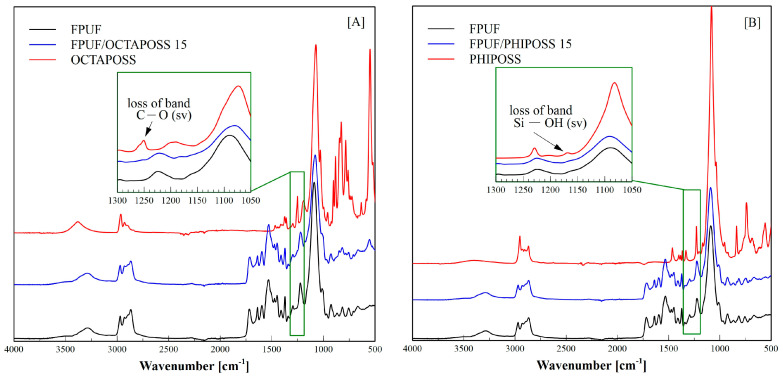
ATR-FTIR spectra of FPUF/OCTAPOSS 15 (**A**) and FPUF/PHIPOSS 15 (**B**).

FT-IR spectroscopy was applied to analyze the structure of the obtained FPU foams (Figure 2).

The FT-IR analysis of the flexible polyurethane foams confirms the reaction of the isocyanate groups, as evidenced by the lack of absorption bands at the wavenumber of 2260 cm^−1^. As a result of the reaction of isocyanate groups with hydroxyl groups, urethane bonds were formed, which was confirmed by the presence of a distinct absorption band on the spectrum at 3285 cm^−1^ resulting from the stretching vibrations of the N-H bonds. The presence of this band shows that practically all N-H groups are linked by hydrogen bonds to the C=O groups of the urethane bond. At the wave number 1715 cm^−1^ and 1640 cm^−1^, there are bands derived from the vibrations of the C=O double bond of the urethane and urea, respectively. A clear signal is visible for the band originating from the stretching vibrations of the C-O-C bonds occurring at the wavenumber of 1089 cm^−1^. At the wavenumber of 1530 cm^−1^, there is a band originating from the stretching vibrations of the C=C bonds of the aromatic ring, which is derived from the aromatic TDI diisocyanate used. In the OCTAPOSS spectrum, there is a band at 1249 cm^−1^ derived from C-O stretching vibrations in the tertiary alcohol group derived from OCTAPOSS. The disappearance of this band in FPUF/OCTAPOSS (Figure 2A) proves the complete reaction of these groups in the polyurethane foam. The presence of POSS is also evidenced by numerous bands appearing in the wavenumber range 600–900 cm^−1^, i.e., the band at 883 cm^−1^ corresponding to the Si-O stretching vibrations, the band at 850 cm^−1^ originating from Si-CH_3_ in OCTAPOSS, and the bands at 755 and 782 cm^−1^ corresponding to the stretching vibrations of Si-C bonds in OCTAPOSS and PHIPOSS. Importantly, the chemical incorporation of PHIPOSS into the PU (Figure 2B) skeleton may be proved by the lack of a specific band at 1170 cm^−1^, corresponding to the stretching vibrations of the Si-OH groups in PHIPOSS.

**Figure 3:**
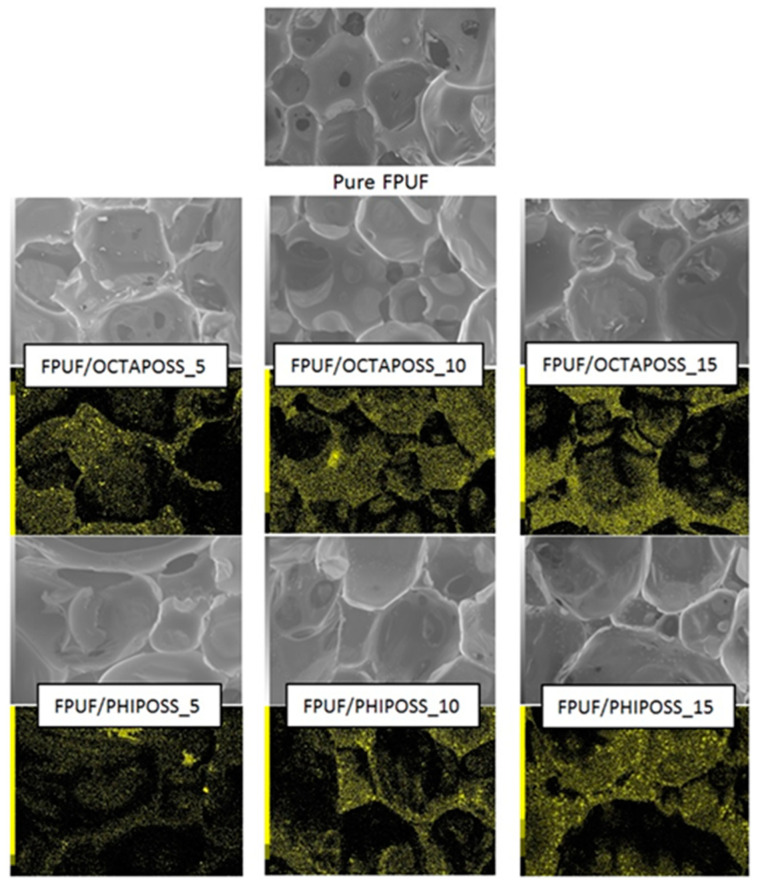
SEM images of pure FPUF and FPUF composites. All images have the same magnification ×80, 100 µm and EDS mapping where show SEI images linked with silicon map.

The cellular structure of the obtained flexible polyurethane foams was evaluated on the basis of the SEM images—Figure 3.

**Figure 4:**
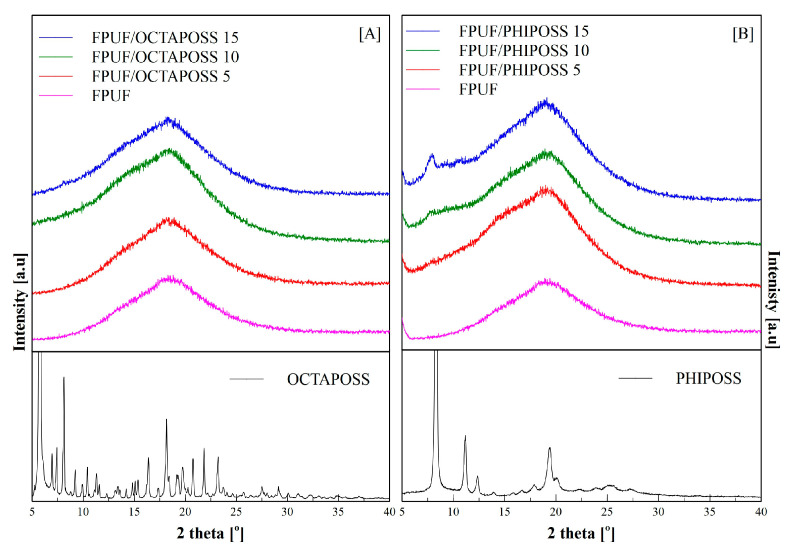
WAXD patterns for pure FPUF, the POSS nanoparticles and FPUF/POSS composites: FPUF/OCTAPOSS (**A**) and FPUF/PHIPOSS (**B**).

The obtained materials have an open cell structure, and the shape of the cells is spherical. It was observed that the addition of POSS to the polyurethane matrix caused a slight increase in the size of the cells. This increase is especially evident for FPUF/PHIPOSS, which may be caused by the formation of PHIPOSS crystallites, which can cause the cells to break and then connect into larger cell structures. Elemental analysis in the form of maps of FPUF/OCTAPOSS materials showed an even distribution of silicon on the surface of the tested material. This is confirmed by the WAXD patterns (Figure 4A). On the other hand, the addition of PHIPOSS to polyurethane foam causes the formation of agglomerates. The greater the proportion of PHIPOSS in the material, the larger the agglomerate clusters. This is also evidenced by the WAXD analysis (Figure 4B), which shows crystallites (peak at 2θ = 8°) derived from PHIPOSS, especially for the FPUF/PHIPOSS 15 sample. Interestingly, in the hexamethylene diisocyanate-based FPUF with POSS [26], a slight tendency of OCTA-POSS to form agglomerates was revealed, whereby numerous PHIPOSS crystals were evenly distributed over the entire surface of the material. It may indicate that the silsesquioxane crystallization behavior in the polyurethane matrix depends on the polymer structure and morphology induced by the kind (aliphatic or aromatic) of diisocyanate.

The WAXD diffractograms of neat PHIPOSS and OCTAPOSS, as well as unmodified FPUF, shown in Figure 4, reveal the difference in the structure of the FPUF/PHIPOSS and FPUF/OCTAPOSS system components. Silsesquioxanes PHIPOSS and OCTAPOSS are high-crystalline substances, as indicated by the numerous sharp diffraction peaks present on the diffraction patterns. On the diffractogram of pure FPUF, only one broad diffuse scattering maxima can be seen, which indicates the existence at the molecular level of only short-range ordering and, thus, the amorphous structure of both the soft and hard polyurethane phases. The neat OCTAPOSS and PHIPOSS show well-arranged crystalline structures represented by sharp peaks in the area starting from 2θ = 5°. An amorphous halo for 2θ = 18.5° from the polyurethane matrix was reported previously [29]. However, despite the highly crystalline structure of OCTAPOSS, foams filled with these nanoparticles only indicated an amorphous structure. This observation is confirmed by SEM analysis which does not show any visible crystalline structures (Figure 4A). Noteworthy, changes in crystallinity were observed in the PHIPOSS-filled foams. Sharp peaks for 2θ = 8° correspond to the arrangements originating from the POSS crystallites. Moreover, a greater concentration of PHIPOSS leads to a higher intensity of the crystalline peaks. The crystallites evidenced at diffraction patterns were also found during SEM analysis as flat white structures located in the cell walls (Figure 4B).

### 3.3. Physicochemical and Mechanical Properties

The apparent density of the foamed polyurethane foams is one of the most important features determining the mechanical properties of foams (Table 2).

The reference flexible foam density was 28.8 kg/m^3^. Both the introduction of OCTAPOSS and PHIPOSS to the foam system increases the apparent density of the obtained materials. However, a greater increase in this parameter was observed for FPUF/PHIPOSS.

For the content of 15% PHI POSS, the apparent density value was 36.3 kg/m^3^, while the same additive content in the case of OCTAPOSS caused an increase in the apparent density of only 32.3 kg/m^3^. The introduction of POSS to the polyurethane system increases the viscosity of the system; therefore, the foaming process may be impeded.

Because POSS also shows a tendency to crystallize, especially PHIPOSS, as shown by the WAXD spectra and the SEM-EDX images, it may cause an increase in the apparent density. This increment correlates with an increase in the number of crystallites formed in FPUF/PHIPOSS compared to FPUF/OCTAPOSS.

The increase in the apparent density of POSS-modified foams is accompanied by improved compressive strength (Table 2). The stress that causes a 40% strain from the original dimension can quantify the compression that the flexible foam supports without significantly losing its morphology. For FPUF, the value of the compressive strength was 12.2 kPa. The introduction of POSS resulted in an increase in the hardness of the foams. In the case of FPUF/OCTAPOSS 15, a greater increase in hardness (24.4 kPa) is observed compared to FPUF/PHIPOSS 15, for which this value was 20.3 kPa. This difference is probably due to the chemical structure of the POSS being introduced. OCTAPOSS has eight functional groups, and in the polyurethane matrix, it is the node of the network, which causes the material to be more cross-linked and, therefore, harder. In contrast, PHIPOSS has two functional groups and forms a branch in the main chain. In addition, it tends to crystallize, which contributes to the formation of larger cells in the foam, and thus the hardness of such a material decreases. Despite the presence of PHIPOSS crystallites, we did not observe the deterioration of the compressive strength parameters compared to the reference polyurethane.

**Figure 5:**
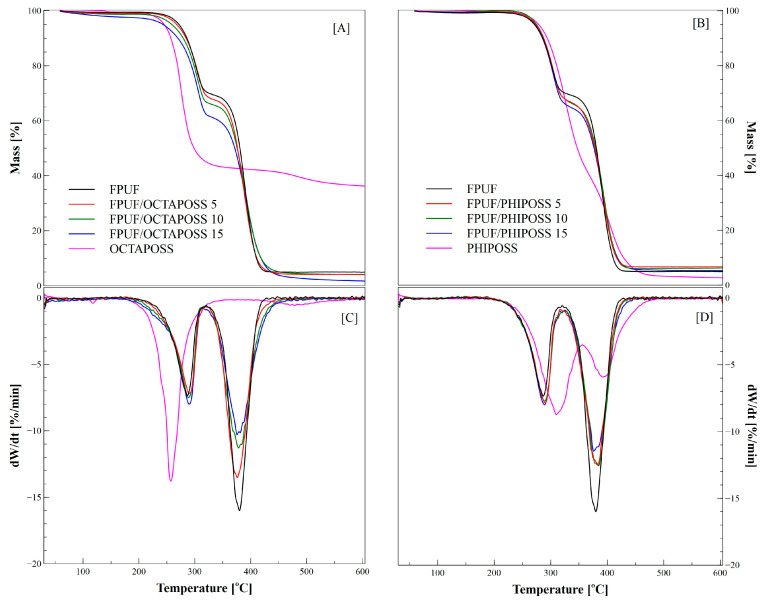
TG (**A**,**B**) and DTG profiles (**C**,**D**) of FPUF/POSS materials under inert atmosphere.

The thermogravimetric analysis allowed for the assessment of the thermal stability of the tested FPUFPOSS foam materials under inert conditions. The TGA plots of POSS degradation are shown in Figure 5. OCTAPOSS degradation (Figure 5A) occurs in two steps. The first takes place at 238 °C, and the weight loss is 57% at 273 °C. This residue degrades slightly between 478 and 600 °C, yielding a final residue of 63% at 600 °C. In the case of PHIPOSS (Figure 5B), the degradation is also a two-step process, the transition between stages is smoother, and the final solid residue is approx. 2%. The stages of degradation of both POSS fillers can be attributed to the partial loss of the organic substituents of the POSS, followed by the formation of the SiOxCy network [30,31].

All of the synthesized polyurethane materials show similar profiles of the thermogravimetric curves, and the thermal degradation of the reference polyurethane and foams with built-in OCTAPOSS or PHIPOSS cages takes place in two separate stages for an inert atmosphere, with two wide peaks in the DTG curves (Figure 5C,D). The degradation of FPUF starts at 259 °C and, in the first step, the urethane bonds in the rigid polyurethane segments are broken down, and in the second step of thermal decomposition, starting at a temperature of about 300 °C, the oligoether diol chains undergo scission. The solid residue for FPUF at 600 °C is 4.9%.

The inclusion of POSS in the EPUF does not significantly modify the thermal degradation path. The first stage of thermal decomposition of the FPUFPOSS is within a narrow temperature range of 259.4–261.8 °C and is characterized by cleavage of the urethane bonds, mainly by depolymerization or rearrangement reactions to TDI and diaminotoluene. The maximum mass losses during the first stage for all samples are comparable and do not depend on the amount of POSS introduced. It can therefore be concluded that, in this case, the presence of silsesquioxane molecules does not significantly slow down the breakdown of the rigid segments. During the second stage of degradation, starting at approx. 300 °C, the oligodiol chains decompose.

At this stage, an intense decrease in the maximum weight loss is observed, along with an increase in the amount of filler introduced. In this case, the DTG_max2_ values (Table 3) vary significantly from −13.5 to −10.3 for FPUF/OCTAPOSS and to a lesser range from −12.5 to −12.0 for FPUF/PHIPOSS. There is also an increase in the solid residue, especially for FPUF/PHIPOSS systems, compared to the reference FPUF. Reducing the maximum rate of weight loss and increasing the solid residue that indicates the presence of a thermally stable inorganic barrier residue from PHIPOSS may be beneficial from the point of view of reducing the flammability of the obtained materials, as it is usually associated with a lower amount of gaseous degradation products released during decomposition, which burn rapidly, causing the fire to spread [26,27,28,31,32].

**Figure 6:**
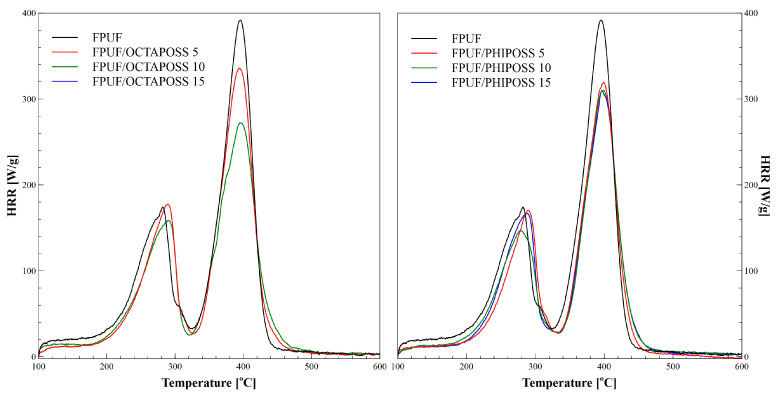
HRR curves of flexible polyurethane foams with POSS.

The method that allows for the definition of the flammability properties of a material is the pyrolysis combustion flow calorimetry (PCFC). It also allows for the characterization of the phenomena accompanying the combustion process. The study of the obtained materials with the use of the pyrolytic combustion flow calorimeter (PCFC) makes it possible to determine the maximum heat of the release rate during the decomposition stages (pHRR), heat release capacity (HRC), and the total heat released (THR). The obtained pyrolysis data are summarized in Table 4 and shown in Figure 6. The analysis of the obtained results shows that the thermal decomposition of the tested materials takes place in two stages, which is consistent with the results obtained by the TG method (Figure 5). The first stage of degradation takes place in the temperature range from 200 °C to 320 °C, and the second stage from 320 °C to 450 °C. Especially in the second stage, a decrease in the pHRR value is observed along with an increase in the amount of introduced POSS in comparison to the reference foam material. This value is the lowest for FPUF/OCTAPOSS 15 and is 228.1 W/g, while for FPUF, it is 387.0 W/g. During the flammability test, the THR value was also measured using the PCFC method, which yields the total amount of heat released by the burning foam. The highest amount of heat—35.2 kJ/g—was released during the testing of the standard foam. The introduction of POSS to FPUF contributed to a significant reduction in the THR value, which did not exceed 29.9 kJ/g. The amount of heat released was the lowest for FPUF/OCTAPOSS 10 and FPUF/PHIPOSS 10. Importantly, these results were obtained with POSS additives only, without the use of any other commonly applied flame retardants, such as phosphonates [26].

The HRC parameter, which characterizes the material’s ability to emit heat during combustion, is considered to be the key parameter determining the reaction of materials to fire [26,27,33]. The HRC value decreases similarly to that of the pHRR. The lowest HRC value among the tested materials is achieved by the FPUF/OCTAPOSS 15 sample, for which the pHRR value is also the lowest compared to the reference system. The results of the PCFC microcalorimetry showed the flame-retardant potential of the obtained modified OCTAPOSS or PHIPOSS materials. This effect may be caused by the specific effects of the POSS used in flexible foam materials. Additionally, the increase in the apparent density of these materials may contribute to reducing the propagation of the flammability process. In addition, POSS in the materials after combustion creates a silica layer, which can also act as an insulating barrier hindering the combustion process.

The Py-GC/MS analysis of POSS nanoparticles and polymer/POSS nanocomposites was studied in the past, e.g., in [34,35,36,37]. The obtained PU-based foam materials containing POSS were subjected to Py-GC/MS analysis, and the results are summarized in Table 5. In the first stage, the side groups from the corners of the silica cage are detached from the structure. When pyrolysis proceeds at a higher temperature, the silsequioxane cage is decomposed. The main products of the pyrolysis of OCTAPOSS nanoparticles are [(1,1-dimethyl-2-propenyl) oxy]dimethyl-silane and allyldimethyl-silanol originating from eight 3-hydroxy-3-methylbutyldimethylsiloxy side groups. A similar effect was observed after the thermal decomposition of PHIPOSS. In the initial stage of pyrolysis, isobutyl side groups detached from the cage corners, forming 2-methyl-1-Propene. At the same time, the functional 1,2-Propanediolisobutyl group disconnects and starts to decompose, forming silica compounds. The presence of carbon dioxide, tetrahydrofuran, and acetone could be a result of consecutive oxidation reactions of the side group decomposition products under the influence of oxygen released from the POSS structure.

The products of the pyrolysis of polyurethane foams can be classified into three groups. The first group includes 1-methoxy-2-propanone,tetramethyl-oxirane, 1,1′-[ethylidenebis(oxy)]bis-propane,1-(1-methylethoxy)-2-propanone,4-methyl-3-heptanol, 3,3′-oxybis-1-propanol, tri(propylene glycol) propyl ether, and 5-methyl-3-heptanol produced from the thermal degradation of the polyether–polyol, followed by dehydration, hydrogen transfer, and ester exchange reactions. The second group of pyrolyzates is compounds formed after the degradation of hard segments containing isocyanates and amines, such as 1,3-diisocyanato-2-methyl-benzene, 7-amino-1,3-dihydro-indol-2-one, and 4-(methyleneamino)phenyldimethyl amine [38]. The presence of isocyanates and carbon dioxide can be a result of the partial depolymerization of urethane bonds. The third group is mainly composed of the products of the pyrolysis of additives. For both OCTA- and PHIPOSS-modified foams, the presence of silicon-containing compounds has been detected. However, 15 wt.% OCTAPOSS foams exhibit higher concentrations of silica derivatives, which could be an effect of the better dispersion of silsesquioxanes in the foams visible on SEM microphotographs or a higher number of silica atoms in OCTAPOSS particles.

## 4. Conclusions

The introduction of POSS to the flexible polyurethane foams influenced the physicochemical and mechanical properties of the hybrid materials. At the synthesis stage, the presence of silsesquioxanes affects the course of the reaction, as evidenced by the dielectric polarization vs. time profiles. A reduced maximum temperature during the foaming process of the hybrids was observed compared to the reference PU foam. For the OCTAPOSS cross-linking agent, core temperature lowering was more intense than for the pendant PHIPOSS modifier. The chemical incorporation of silsesquioxane into the polyurethane structure was confirmed by IR analysis, and elemental analysis maps of FPUF/OCTAPOSS materials revealed an even distribution of silicon on the surface of the tested material. Moreover, the WAXD patterns show only amorphous structures. In foams containing PHIPOSS, some agglomerates were detected, which may be associated with the local accumulation of pendant silsesquioxane cages. The good dispersion of OCTAPOSS particles in the polyurethane matrix caused an increase in the compressive strength of the composites, especially for the material with the highest POSS load. Py-GC/MS analysis allowed for the detection of the main products of the thermal decomposition of the modified foams formed via chain scission or depolymerization reactions, as well as by dehydration. POSS moieties degrade in a two-step process: (i) detachment of organic functional groups and (ii) the decomposition of the Si-O cage structure to form silica residue—the latter supports the formation of a thermal barrier on the materials’ surface. The applied POSS acted as flame retardants and lowered the THR and pHRR parameters in the cone calorimeter tests. The analysis of flammability data showed that the modified flexible polyurethane foams are less prone to fire development compared to the reference foams. POSS during combustion creates a silica layer, as evidenced by Py-GC/MS analysis, which can act as an insulating barrier hindering heat and mass transport.

## Figures and Tables

**Table 1:** Formulation of flexible polyurethane foams modified by POSS.

Reagents	FPUF	PHIPOSS			OCTAPOSS		
Polyol [%]	100						
PHI-POSS [%wt.]	-	5	10	15	-	-	-
OCTA-POSS [%wt.]	-	-	-	-	5	10	15
Water [%]	4						
Surfactant [%]	1.5						
Catalyst [%]	1.0						
TDI, NCO index	1.0						

**Table 2:** Apparent density and compression strength of FPUFs.

Measurement	FPUF [%]	FPUF/PHIPOSS [%]			FPUF/OCTAPOSS [%]		
		5	10	15	5	10	15
Apparent Density [kg/m^3^]	28.8 ± 0.1	32.6 ± 1.4	33.7 ± 1.6	36.3 ± 1.5	30.7 ± 1.4	31.4 ± 1.6	32.3 ± 1.4
Compression Strength [kPa]	12.2 ± 2.3	14.4 ± 3.1	14.4 ± 0.8	20.3 ± 3.9	20.3 ± 2.2	24.0 ± 0.9	24.4 ± 1.9

**Table 3:** TG results of polyurethane foams with POSS (in inert atmosphere).

Samples	T_onset_ [°C]	ΔT_onset_ [°C]	T_DTGmax1_ [°C]	T_DTGmax2_ [°C]	DTG_max1_ [%/min]	DTG_max2_ [%/min]	m_1_ [%]	Residual Mass [%]
FPUF	259.5	-	286.8	379.8	−7.3	−16.0	30.6	4.9
FPUF/OCTAPOSS 5	260.7	1.2	290.1	376.0	−7.2	−13.5	31.3	4.1
FPUF/OCTAPOSS 10	259.5	0.0	288.8	378.1	−7.5	−11.3	32.8	4.1
FPUF/OCTAPOSS 15	259.4	0.1	290.0	375.6	−3.1	−10.3	36.2	1.7
FPUF/PHIPOSS 5	261.8	2.3	290.2	382.8	−7.9	−12.5	32.7	6.6
FPUF/PHIPOSS 10	260.9	1.4	289.4	384.6	−7.7	−12.5	33.8	6.2
FPUF/PHIPOSS 15	261.1	0.6	288.9	376.0	−7.7	−12.0	35.5	5.2
OCTAPOSS	237.8	−21.3	257.3	478.5	−13.8	−0.6	57.3	36.3
PHIPOSS	272.4	12.9	312.3	393.7	−8.6	−5.9	60.1	2.7

**T_onset_**—extrapolated temperature of the beginning of thermal decomposition, **ΔT_onset_**—temperature difference for the beginning of thermal decomposition, **T_DTGmax1_**, **T_DTGmax2_**—temperatures of maximum rate of residual mass for the first and second stage, **DTG_max1_**, **DTG_max2_**—maximum rates of residual mass for the first and second stage, **m_1_**—residue mass for the first stage of thermal degradation.

**Table 4:** Test results for flexible polyurethane foams on a pyrolysis combustion flow calorimeter.

Sample	pHRR_2_ [W/g]	THR [kJ/g]	HRC [J/gK]	T_2_ [°C]
EPUF	387.0 ± 29.0	35.2 ± 2.8	419 ± 28	395 ± 4
EPUF/OCTAPOSS 5	323.1 ± 11.0	31.7 ± 2.7	351 ± 12	395 ± 3
EPUF/OCTAPOSS 10	261.4 ± 15.1	29.9 ± 1.2	277 ± 17	388 ± 9
EPUF/OCTAPOSS 15	228.1 ± 9.2	30.2 ± 0.8	248 ± 12	387 ± 3
EPUF/PHIPOSS 5	314.7 ± 16.4	31.1 ± 2.9	342 ± 21	399 ± 1
EPUF/PHIPOSS 10	310.0 ± 14.6	29.9 ± 0.9	337 ± 18	396 ± 1
EPUF/PHIPOSS 15	307.9 ± 21.4	31.3 ± 2.1	333 ± 24	396 ± 2

**pHRR_2_**—peak of heat release rate; **THR**—total heat released; **HRC**—heat release capacity; **T_2_**—temperature at pHRR.

**Table 5:** Pyrolysis products of flexible polyurethane foams containing POSS.

Compounds	Retention Time [min]	Identified				
		OCTAPOSS	PHIPOSS	FPUF	FPUF/ OCTA POSS 15	FPUF/ PHI POSS 15
Carbon dioxide	1.2		x	x	x	x
2-methyl-1-Propene	1.7		x			
Acetone	1.8		x			
Propene	1.9			x	x	x
1-methoxy-2-propanone	2.0			x	x	x
Tetrahydrofuran	2.3		x			
Tetramethyl-oxirane	2.8			x		
[(1,1-dimethyl-2-propenyl)oxy]dimethyl-silane	3.7	x			x	x
1,1′-[ethylidenebis(oxy)]bis-propane	3.8			x	x	x
1-(1-methylethoxy)- 2-propanone	4.0			x	x	x
Allyldimethyl-silanol	5.4	x			x	x
4-methyl-3-heptanol	5.6			x	x	x
3,3′-oxybis-1-propanol	5.8			x	x	x
3-Buten-1-ol TBDS derivative (isomers)	6.6		x			
tri(propylene glycol) propyl ether	7.3			x	x	x
5-methyl-3-heptanol	7.4			x	x	x
1,3-diisocyanato-2-methyl-benzene	9.1			x	x	x
7-amino-1,3-dihydro-indol-2-one	9.5			x	x	x
4-(methyleneamino)phenyldimethyl amine	9.6			x	x	x

## Data Availability

The data presented in this study are available on request from the corresponding author.
